# Knowledge and Awareness of Masks and N95 Respirators Used for COVID-19 Prevention Among Chemical Engineering Students at Al-Balqa Applied University, Jordan

**DOI:** 10.3389/fpubh.2021.620725

**Published:** 2022-01-07

**Authors:** Banan Hudaib, Ali F. Al-shawabkeh, Fadia Hudaib

**Affiliations:** ^1^Chemical Engineering Department, Faculty of Engineering Technology, Al-Balqa Applied University, Amman, Jordan; ^2^Physics and Basic Sciences Department, Faculty of Engineering Technology, Al-Balqa Applied University, Amman, Jordan; ^3^Amman College of Banking and Financial Studies, Al-Balqa Applied University, Amman, Jordan

**Keywords:** COVID-19, masks and respirators, chemical engineering students, Jordan, Al-Balqa Applied University

## Abstract

**Background and Objectives:** On March 11, the World Health Organization stated coronavirus disease 2019 (COVID-19) was a global pandemic; the rapid and extended spread of the COVID-19 pandemic has become a significant cause of concern for face-to-face university study. This study investigated the knowledge and awareness of chemical engineering students in Al-Balqa Applied University (BAU) in Jordan about respiratory protective measures against COVID-19.

**Materials and Methods:** A cross-sectional study was developed using a multi-stage random sampling technique conducted from April 21–28, 2020. The data were collected through an online questionnaire distributed to BAU's chemical engineering students, with 179 responders completed the survey correctly. The data were analyzed statistically using the SPSS program. The questionnaire consisted of two parts: the first measured the student's general knowledge about the COVID-19 pandemic, while the second focused on the respiratory protection methods against COVID-19; it was structured to assess the student's knowledge about the suitable types of masks and respirators used in COVID-19 prevention, their detailed mechanism of action and filtration process type, their production materials, and finally how the student's knowledge affects choosing a proper preventive method.

**Results:** The study found moderate awareness among engineering students about COVID-19 causative agent, effective preventive masks/respirators used, and the mask's viral blockage mechanisms. A total of 89 respondents (49.7%) pointed to the correct best protective mask, i.e., N95 mask. On the other hand, 119 respondents (66.5%) believed that a surgical mask is the best protective mask. The study also showed differences in knowledge between different academic years; the knowledge about respirators, masks, and their action mechanism among senior students in the last three academic years was better than the younger students with a *P*-value of 0.047 for knowledge about respirator used for protection against coronavirus disease and the *P*-value of 0.028 for knowledge of the comparisons between the N95 and surgical mask. On the other hand, the study showed a lack of awareness of the most suitable mask types used in pandemics and the appropriate use method.

**Conclusions:** The study found that chemical engineering students in Al-Balqa university were moderately knowledgeable regarding COVID-19 respiratory preventive methods; these results provided an overview of each student's community's knowledge level. Therefore, efforts are needed to improve public awareness through comprehensive educational campaigns to increase students' knowledge, attitude, and practice.

## Introduction

Infectious diseases are considered to be the second leading cause of death worldwide (1). Among infectious diseases causing death worldwide, acute lower respiratory tract infections, HIV/AIDS, and diarrheal diseases predominated (2, 3).

In late December 2019, multiple influenza and severe pneumonia cases spread quickly in Wuhan, China, and a new species of the coronavirus family was responsible for the new zoonotic disease (4). Initially, the World Health Organization (WHO) named the causative virus as 2019 novel coronavirus (2019-nCOV), and then the name was updated later to SARS-COV-2 (5), and the disease was named coronavirus disease 2019 (COVID-19) (6, 7). On March 11, 2020, WHO declared COVID-19 as a global pandemic, and the new disease started to spread worldwide in many countries, including Jordan (8).

COVID-19 is a respiratory syndrome, and it is of paramount importance to determine the route of transmission of the infectious agent (e.g., directly via respiratory droplets/airborne or indirectly through contaminated, infected surfaces) to build a suitable strategy to cope with a pandemic which will be based solely on this knowledge and upon the preventive tools available (9).

The primary disease spread prevention strategies in a new influenza pandemic rely solely on non-pharmacological primary public health measures as a part of a larger strategy to establish barriers and increase distancing between infected and uninfected individuals (10), as the lack of the effective vaccine and antiviral medications and the long time required to develop them make these the available defense measures (11).

The awareness of the importance of respiratory barriers in preventing respiratory disease transmission, knowledge of the general differences between the different types of respirators and masks used in the COVID-19 pandemic, and the type of filter (membrane) used in these masks had a significant influence on the personal attitude toward the use of these barriers (12). Masks are routinely and obligatorily used by chemical engineers in the work environment to deal with different chemicals and gases. Chemical engineering students are studying membranes, filtration, and separation processes topics during the Bachelor of Science curriculum starting from the third year.

Therefore, our study aimed to assess knowledge and awareness among our chemical engineering students regarding the types of respirators, mask, and the significant differences between various types, as well as to measure the extent of their knowledge about the filtration mechanism and the impact of the student's knowledge on the choice of the appropriate mask to prevent COVID-19. In addition, the study aimed to assess the knowledge effect on understanding the best preventive measures of pandemic transmission and explore the differences in knowledge between junior and senior students.

## Theoretical Background and Literature Review

COVID-19 virus, which belongs to a big family of coronaviruses that causes different infectious diseases, is the most recently discovered coronavirus family member. The new virus was unknown before the outbreak started in Wuhan, China, in December 2019. However, the enormous and rapid outbreak of COVID-19 affected most world countries leading to the WHO declaration of COVID-19 as a global pandemic.

COVID-19 spread mainly from an infected person to others by breathing the expelled tiny droplets from the nose or mouth during coughing, sneezing, or speaking. Those droplets are relatively heavy and quickly dropped to the ground's surface; thus, people can become infected by touching these objects or surfaces. Therefore, wearing masks, wearing gloves, and washing hands regularly are the primary protection methods (13, 14).

According to the WHO, there are three main types of masks used to protect against the spread of COVID-19. Those are (15):

1. Medical masks or surgical masks made from a minimum of three layers of synthetic non-woven materials and configured to have filtration layers sandwiched in the middle.
2. Non-medical masks (fabric masks, homemade masks, DIY masks) act as a barrier to prevent virus spread from the wearer to the public. Numerous types of fabric masks are not standardized, and other preventive measures should be taken.
3. Respirators, or filtering face-piece respirators (FFP), are specifically designed for healthcare workers (HCWs) providing care to COVID-19 patients in hospitals and areas where aerosol-generating procedures are performed. These respirators are available at different performance levels, such as FFP2, FFP3, N95, and N99. Healthcare workers should be fit tested before using a respirator to ensure they are wearing the correct size (16).

Alzoubi et al. (6) evaluated the COVID-19 knowledge, practice, and attitude among Jordan's medical and non-medical university students. The study's findings showed a positive response regarding the overall knowledge about the symptoms of COVID-19. All students agreed that hand washing is necessary to prevent infection, whereas 68.4% believed that masks prevent the infection. One-fifth of them thought that antibiotics and smoking were protective measures against infection. Concerning avoiding handshaking, washing hands, using alcoholic hand sanitizer, coughing, or sneezing in tissue and disposing of it, almost all students agreed on these preventive practices. In this study, social media, the internet, and television were the primary sources of students' knowledge about COVID-19. The results showed no significant differences in knowledge between medical and non-medical students.

Modi et al. (11) studied COVID-19 awareness among healthcare students and professionals in Mumbai metropolitan region and found that the overall awareness for all subgroups was adequate. A total of 71.2% of respondents reported correct answers in the Mumbai metropolitan region. Undergraduate medical students gave the highest percentage of correct responses, while the lowest was the non-clinical administrative staff. The results showed that less than half of the total respondents correctly defined “close contact.” At the same time, more than three-fourths of the respondents were aware of the various infection control measures like rapid triage, respiratory hygiene, cough etiquette, and having a separate, well-ventilated waiting area for suspected COVID-19 patients. Nevertheless, only 45.4% of the respondents knew the correct sequence for applying a mask/respirator, and only 52.5% of the respondents were aware of the preferred hand hygiene method for visibly soiled hands.

Kumar et al. (7) investigated HCW's knowledge, attitude, and practices regarding using face masks for COVID-19 prevention. They found that 43.6% of participating HCWs in Pakistan knew the correct method of wearing the masks, 70% knew the number of layers, 53% stated that the middle layer acts as a filter media barrier, and more than three-fourths knew the recommended maximum wearing duration. The results also showed that the majority, 88.2%, of participants knew that a cloth face mask is less effective, and 79.8% knew that worn face masks should not be re-used, while 44.8% knew about the yellow coded bag for disposal. Although the study concluded that the knowledge, attitude, and practice of HCWs regarding the use of face masks were inadequate, it was noticed that there is a positive attitude, but a moderate-to-poor level of knowledge practice, regarding face masks use among HCWs. The study recommended that awareness campaigns for HCWs and the general public regarding the proper use of face masks utilizing all social media resources can help.

Iboi et al. (12) investigated the rule of effective public health education measures in minimizing both the cumulative and daily mortality of the COVID-19 in the United States. The results indicated that more coronavirus cases were experienced in places with a reduced commitment to public health measures, and the daily mortality was increased compared to places with strict commitment. Similar results were obtained by other studies (10, 17), where the universal use of face masks significantly reduced community transmission of COVID-19 and brought the pandemic under effective control. This highlights the importance of knowledge about mask types, appropriate mask selection, and the protection mechanism of the most suitable masks against COVID-19.

Iboi et al. (12) carried out a modeling study to determine the influence of public health education programs on the US coronavirus outbreak. The model used cumulative mortality data for the United States from January to December 2020. According to the study, a 10% increase in education rate from the baseline reduces the peak daily mortality by 66.3, 26.7, and 16% by April, July, and December, respectively, compared to a baseline scenario. However, a 40% increase in education from the baseline reduces the peak daily mortality by 96.8, 44.5, and 54.8% by April, July, and December, respectively. This demonstrated improved public education and the universal use of face masks curtailed COVID-19 transmission and effectively affected the pandemic.

### The COVID-19 Outbreak Worldwide and in Jordan

According to the last statistics of the Worldometer website, a total of 237,826,700 registered cases infected with COVID-19 (214,870,766) recovered, and the virus caused the death of about 4,853,179, representing about 2% of the closed cases (18).

In Jordan, 831,832 infected cases were registered with 10,789 deaths, and 807,569 cases recovered.

**Figure 3** shows the trends of the new and accumulated cases globally and in Jordan.

### Using Masks in the Context of COVID-19 Worldwide

**Figure 1:**
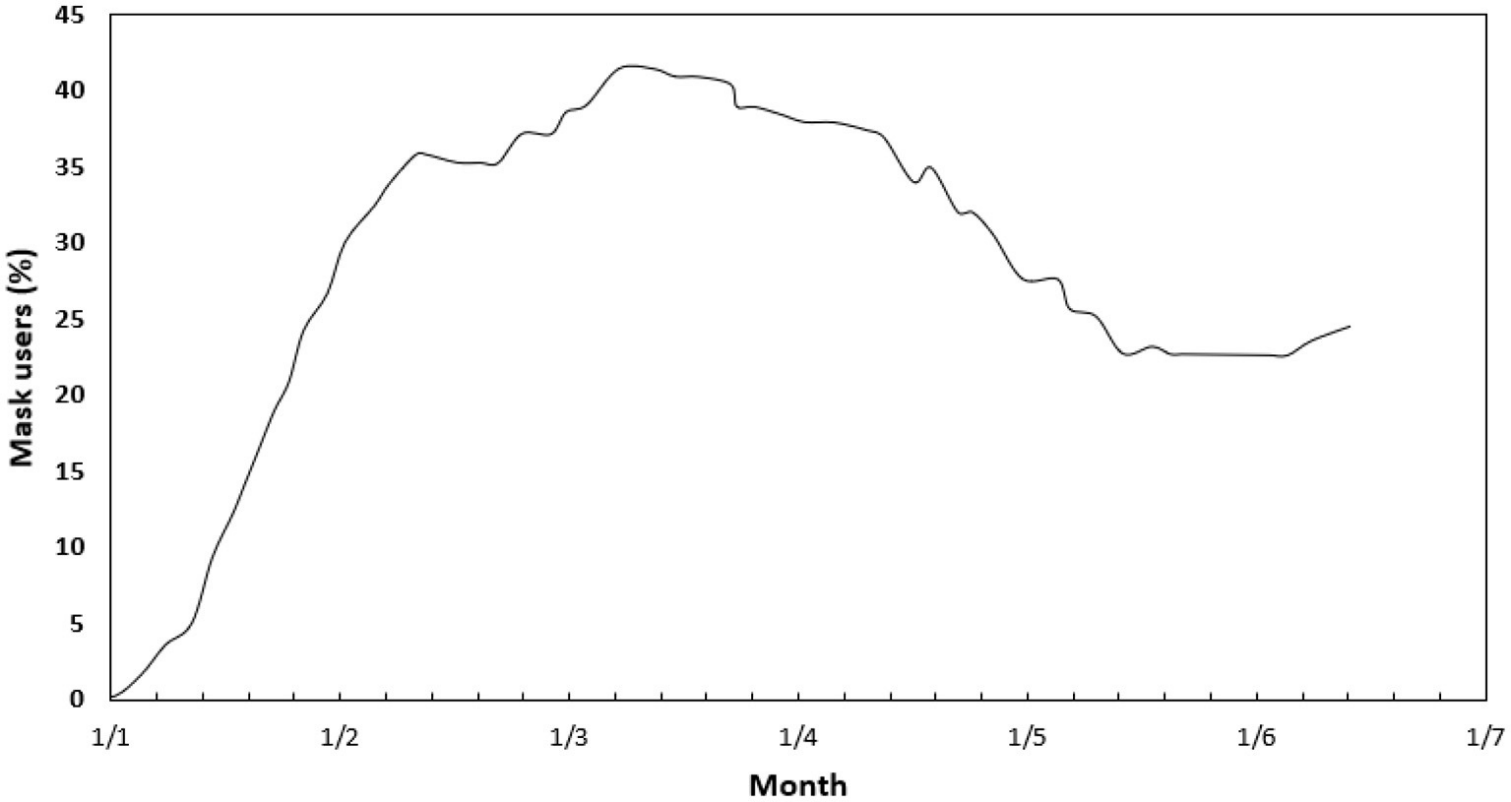
The projected masks users percentage in Jordan.

**Figure 2:**
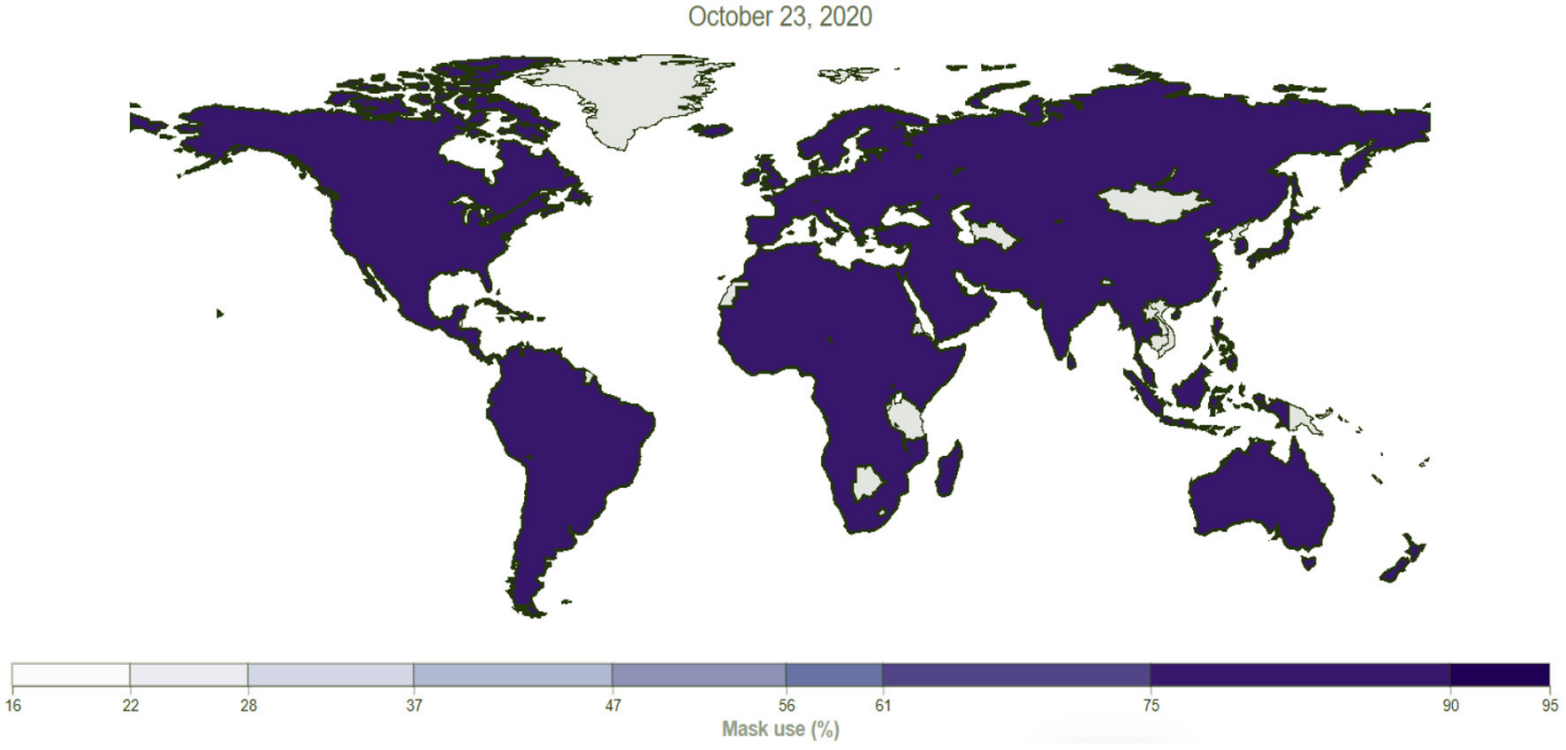
The percentage of mask users of the population in the world. Data sources: https://covid19.healthdata.org/jordan?view=mask-useandtab=trend, (22, 28).

**Figure 3:**
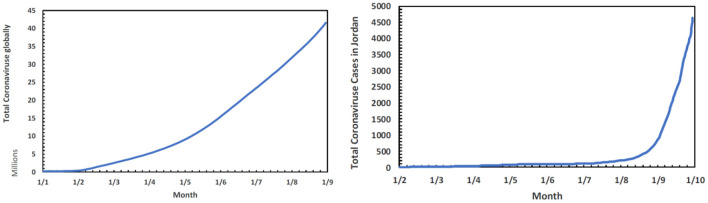
Trends of the new and accumulated cases globally and in Jordan.

One observational study showed that using face masks was 79% effective in preventing COVID-19 transmission if all members applied them before symptoms appeared (19). A survey done by Ipsos group (20). in April 2020 showed that among people across 15 major countries, the highest percentage of people who wore masks were in Vietnam (91%), China (83%), Italy (81%), Japan (77%), and India (76%), and the high percentage in Vietnam was attributed to the high percentage of survey respondents who believed that people should wear masks in public to protect others (21). Figures 1, 2 shows the mask user's percentage in Jordan and worldwide, respectively. Figure 2 illustrates that 24% of Jordan's population said they always wear a mask in public compared to the universal target, which is 95%. However, compared to other countries worldwide, Figure 3 indicated that the population who used masks is <54% in most countries, including the United States, Europe, and Russia. In contrast, the highest percentage was in Mexico, the western countries of South America, Saudi Arabia, and South Africa countries, in which the percentage exceeded 74% of the total population.

## Methodology

A cross-sectional study was conducted using a multi-stage random sampling technique from April 21–28, 2020. The data were collected through an online questionnaire distributed to the chemical engineering students of Al-Balqa Applied University (BAU). The target population for this study is 400 students enrolled in the BAU chemical engineering department in Amman. The questionnaire was distributed to the students through the internet (department Facebook groups). Out of 400 enrolled students in the department, 179 responders completed the survey correctly, the author received 200 responses and excluded 15 incomplete responses and six responses filled by non-chemical engineering students. The data were analyzed statistically using the SPSS software. The online developed questionnaire consisted of a 20-paragraph/multiple-choice designed in two parts to fulfill the study objectives. The first part is about the respondent's demographic characteristics (age, level, gender). The second part measured the respondent's knowledge about COVID-19, transmission and prevention methods, and the quality of masks and respirators used. Moreover, a two tail *T*-test was used to explore whether any statistical difference existed in the respirator knowledge between students of the first 2 years (group 1) and the last three academic years (group 2) using SPSS for the analysis.

The validity of research findings was ensured through expert consultations with lecturers from BAU. According to Borg and Gall (23), the content validity of an instrument is improved through expert judgment. To enhance the reliability of the study findings, piloting of the questionnaires was conducted among selected chemical engineering students in BAU. The piloting sample size was selected on a proportion of 10% of the study sample size and then the reliability of the study instruments was calculated using Cronbach's Coefficient Alpha. According to Fraenkel and Wallen (24), a reliability coefficient of 0.7 and above implies that the study instruments are reliable; thus, this criterion was used to assure the reliability of the study instruments.

## Results and Discussions

### Source of Information About COVID-19 Among Students

Table 1 shows the demographic characteristic of the respondents.

**Table 1:** The demographic characteristic of the study sample.

**Variable**		**Frequency**	**Percentage**
**Gender**	Female	103	57.5
	Male	76	42.5
**Age**	18–25	164	91.6
	26–30	10	5.6
	31–35	5	2.8
**Academic year:**	Fresh (1st year)	8	4.5
	Sophomore (2nd year)	59	33.0
	Junior (3rd year)	16	8.9
	Seniors (4th year)	54	30.2
	Super Senior (5th year)	42	23.5
**Total respondents**		179	100.0

Out of 400 enrolled students in the department, 179 respondants (44.7%) completed the survey correctly; the author received 200 responses and excluded 15 incomplete responses and six responses sent by non-chemical engineering students. The female respondants in the study sample were 103 (57.5%), and the rest were males. Most of the respondents were between 18–25 years old. Fifty-nine students (33%) were second-year level, 54 (30.2%) and 42 students (23.5%) were fourth- and fifth-year levels, while first- and third-year level students made only 8 (4.5%) and 16 students (8.9%), respectively. As there is an optional course for membrane and separation processes offered to 3rd, 4th, and 5th-year students, most of the respondents from this academic level are expected to be familiar with different types of masks, their mechanism of action, and their appropriate use, and this knowledge would help to answer the questionnaire.

Table 2 showed that social media was the primary source of information about COVID-19; 178 students (99.4%) knew about the pandemic from social media as a single source or in addition to other sources. TV/Radio was the second source of information with 111 students (62.3%), followed by 65 students (36.3%) knew from friends and colleagues. Also, 31 students (17.3%) mentioned that the source of information about COVID-19 was posters/brochures and 36 students (20.3%) learned about COVID-19 from seminars.

**Table 2:** Sources of Knowledge about COVID-19 outbreak.

**Method**	**Percentage**
Social media	99.4
TV/radio	62.6
Poster and brochures	17.3
Friends and colleagues	36.3
Seminars	20.1
One method	22.9
Two methods	43
Three methods	10.1
Four methods	14.5
Five methods	7.3
	100.0

Forty-one students (22.9%) mentioned social media as the only source of information about COVID-19, which points to the importance of social media as an essential source of information in the COVID-19 pandemic.

### Knowledge About the Disease

Knowledge about the disease is fundamental for protecting ourselves and making the most effective measures to prevent the disease.

Most of the respondents thought that COVID-19 is a serious public health problem; however, only 38 students (21.2%) correctly named the causative agent, i.e., the SARS-Cov-2 virus. It is well-known that contact with an infected person is the primary source of infection of COVID-19 either through breathing the droplets from the nose or mouth expelled when the infected person coughs, sneezes, or speaks from a distance <1 m, or through touching contaminated surfaces (25). A total of 126 respondents (70.4%) pointed to those two methods of COVID-19 transmission in addition to one or more other methods. However, 185 respondents (88.3%) mentioned contact with an infected person besides other methods, and 146 (81.6%) mentioned touching contaminated surfaces besides other methods.

The previous results strongly indicated an excellent general knowledge among students regarding disease transmission methods.

Table 3 shows that most of the respondents mentioned more than one method of virus prevention. A total of 112 respondents (62.6%) mentioned masks and 121 respondents (67.8%) mentioned face shields, and 68 respondents (38%) chose both of them as an efficient method of preventing disease besides other methods, which indicates the respondents' awareness of mask and face shield importance in preventing COVID-19 infection.

**Table 3:** The attitudes of the respondents about COVID-19 knowledge about protective mask used in COVID-19.

		**Percentage**
**The risk of COVID-19**	Serious public health problem	95.5
**Coronavirus disease COVID-19 is caused by:**	MERS-CoV	2.2
	Bacteria from bats	16.8
	MERS-CoV	8.4
	SARS-COV2	21.2
	No idea	51.4
**Coronavirus disease (COVID-19) is transmitted by**	Coughing and sneezing	82.1
	Air	34.6
	Handshaking	65.9
	Contact with an infected person	88.3
	Touching contaminated surfaces	81.6
	Food	44.7
	Contact with an infected person and Touching contaminated surfaces	70.4
**Coronavirus disease (COVID-19) can be prevented by**	Avoiding crowds	81.3
	Social distancing	61.5
	Gloves	77.1
	Masks	62.6
	Face shield	67.8
	Both masks and shield	38.0
	Avoiding traveling	78.2
	Staying home	78.8
	More than one	96.1

Most respondents stated that using masks, wearing gloves, and washing hands prevents the disease's transmission effectively.

#### Quality of Protection Masks

Table 4 shows students' responses regarding the details of the masks used to protect against COVID-19. The table indicates that 85 respondents (47.5%) knew the filter type and understood its action mechanism and how it affected the selection of the mask type; this may be attributed to their academic background and familiarity with masks in the lab. However, the respondents who answered with no idea are very high for most of those questions.

**Table 4:** Knowledge about the respirators used for protection against Coronavirus disease (COVID-19).

**Understanding of filter type and mechanism affect our attitude in the selection of the mask type**	**Yes**	**47.5**
**Types of masks/respirators**	Surgical mask	66.5
	N95 mask	49.7
	P95 mask	29.6
	Scarf	58.1
	No idea	44.1
**Material for membranes**	Non-woven fabrics	51.4
**used in the filter of**	Silk	6.1
**masks/respirators**	Wool	3.9
	No idea	38.5
**Type of the membrane filtration**	Between ultrafiltration and microfiltration	19.6
	reverse osmosis	6.7
	Between reverse osmosis and nanofiltration	17.3
	No idea	56.4
**In using respirators/surgical**	Sterilize	24.0
**masks. It is recommended**	Avoid wearing mask	7.3
**to:**	Dispose of after use	34.6
	Use it continuously	12.8
	No idea	31.3
**The mechanisms in which**	Interception of the virus	20.7
**the masks/respirators block**	Inertial blockage of the virus	30.7
**viruses**	Diffusion	26.3
	Electrostatic	5.6
	No idea	45.3
**The filtration process in**	Mechanism	36.9
**respirators and masks**	Number of mask layers	18.4
**depends on**	Type of polymers used	20.1
	Size of the filtered	26.3
	No idea	39.7

Table 4 showed that 89 respondents (49.7%) chose the correct best protective mask, i.e., N95 mask (10, 16). In contrast, about two-thirds of the respondents believed that the surgical mask was the best protective mask. In addition, 104 students (58.1%) mentioned the scarf, and 79 students (44.1%) had no idea about the correct type of mask that should be used.

Moreover, 92 respondents (51.4%) pointed to non-woven fabrics as the correct material used in masks/respirators production. Only 35 respondents (19.6%) knew the correct membrane filtration process used in masks (26). A total of 62 respondents (34.7%) stated that the mask should be disposed of after a single use.

A total of 37 students (20.7%) mentioned that the mechanisms by which the masks/respirators can block viruses are interception, while 55 students (30.7%) chose inertial blockage, and 47 students (26.3%) chose diffusion (27).

Table 4 shows that 66 students (36.9%) stated that the factor affecting filtration process in respirators and masks is the mechanism of filtration, while 32 students(18.4%) chose the number of mask layers and 35 students (20.1%) chose the type of polymers used, while the size of the filtered material was chosen by 47 students (26.3%) (28).

Although the study population presumed to have good academic background regarding membranes and filtration processes, the results showed that this academic background is inadequate, with only half of the respondents knowing the correct mask type and its blockage mechanism.

Table 5 reflected students' understanding of differences between various protective masks; more than half of the respondents realized that surgical masks are a more practical choice in the COVID-19 pandemic than N95. However, only two students (1%) knew that N95 masks are more efficient than surgical masks, and 27 students (15%) knew that the N95 mask was named after a blockage of (95%) of aerosols particles.

**Table 5:** Comparison between the N95 and surgical masks.

**Regarding the differences between surgical masks and respirators (N95)**	**N95 is used to reduce the risk of inhaling hazardous airborne particles**	**35.2**
	Surgical masks are designed to be used by an infected person, healthcare worker, or member of the public to reduce the transfer of body fluids that may spread infection	9.5
	Both are the same	6.1
	N95 are more efficient than surgical masks	1.1
	Surgical masks are a more practical choice in pandemic	53.6
**N95 respirators**	Block 5% of the aerosol's particles	12.3
	Used for an oil-free atmosphere	6.7
	Block 95% of the aerosol's particles	15.6
	Collect all particle size >95%	21.2
	No idea	56.4

#### The Authority Responsible for Testing and Approval of N95/P95 Respirators

Table 6 indicates that 101 respondents (56.4%) agreed that National Institute for Occupational Safety and Health (NIOSH) is the authority responsible for testing and approval of N95/P95 masks (16). National Institute for Occupational Safety and Health, a part of the U.S. Centers for Disease Control and Prevention, certifies filtering face-piece N95 respirators that meet the criteria for a minimum 95 filter efficiency at the most penetrating particle size. While unfortunately, most respondents were not aware of the authority responsible for surgical masks testing, only seven students (3.9%) knew that Food and Drug Administration (FDA) is responsible for testing and approving surgical masks (16).

**Table 6:** Authority responsible for testing and approval of N95/P95 respirators.

**Organization**	**N95/P95**	**Surgical masks**
**FDA**	19.6	3.9
**WHO**	6.7	25.1
**NIOSH**	56.4	20.1
**NO IDEA**	17.3	50.8
	100.0	100.0

Table 7 demonstrates the statistical comparison between students of the first 2 years (group 1) and the last three academic years (group 2) using SPSS, two tail *T*-test regarding knowledge and sources of knowledge about COVID-19, the respondent's attitudes about COVID-19, knowledge about the respirators used for protection against COVID-19, and comparison between the N95 and surgical masks. The *p*-values of knowledge, sources of knowledge and attitude toward COVID-19, knowledge about the respirators, and N95/surgical masks comparison were 0.345, 0.317, 0.047, and 0.028, respectively. According to two tailed-values (as the *p*-value >0.05) there were no significant statistical differences between the students regarding knowledge, source of knowledge, and attitude toward COVID-19 based on their academic year; these results are expected due to global-wide coverage of disease facts in social media and the strict measures imposed by the governments immediately after its announcement as a pandemic by WHO.

**Table 7:** Independent Samples Test, regarding the mean difference of knowledge and source of knowledge, attitude, knowledge about the respirators and comparison between the N95 and surgical masks level between chemical engineering students of the first three academic years and the last two academic years.

	***T***	***df***	**Sig (2-tailed)**	**Mean difference**
**Knowledge and sources of knowledge about coronavirus disease (COVID-19) outbreak**	−0.686	112	0.345	−0.008
**The attitudes of the respondents about the coronavirus disease**	−0.611	112	0.317	−0.002
**Knowledge about the respirators used for protection against coronavirus disease (COVID-19)**	−1.516	175	0.047	−0.017
**Comparison between the N95 and surgical masks**	−1.591	175	0.028	−0.022

There were significant statistical differences between the two groups regarding knowledge about the respirators used for protection against COVID-19 and the differences between N95 and surgical masks, with *P*-value <0.05. These results could be explained, as group 2 (senior students) have finished many topics related to the separation processes in their college curriculum.

### Limitations of the Study

The limitations of this study could include a low response rate and small sample size, which could be due to lockdown during the survey distribution time and sampling-related issues like the inability to verify the respondent's identity. However, the questionnaire can be extended to include the entire engineering students at Al-Balqa University and several universities in Jordan to investigate the knowledge trends among engineering students in Jordan. This survey was done in the early time of COVID-19. This could be added to limitations as the strategy and knowledge regarding COVID-19 were still growing among the public worldwide. Hence, the results would be different 1 year later.

## Conclusion

This study showed moderate awareness of engineering students in BAU-Jordan about masks used in COVID-19 prevention. Keeping in mind the students' academic background regarding membranes and separation process, it is evident that the knowledge of membrane type and filtration process and blockage mechanism was inadequate, adversely affecting students' choice of suitable preventive masks. Therefore, it is recommended to increase the number of academic courses focusing on membranes and filtration processes; this will help raise knowledge and awareness and engage students positively in changing community COVID-19 prevention attitude.

## Data Availability Statement

The raw data supporting the conclusions of this article will be made available by the authors, without undue reservation.

## Ethics Statement

Ethical review and approval was not required for the study on human participants in accordance with the local legislation and institutional requirements. Written informed consent for participation was not required for this study in accordance with the national legislation and the institutional requirements.

## Author Contributions

BH prepared the survey and collected the data. AA-s and FH contributed to statistical analysis of the data. BH, AA-s, and FH authors involved in the discussion of the results. All authors contributed to the article and approved the submitted version.

## Conflict of Interest

The authors declare that the research was conducted in the absence of any commercial or financial relationships that could be construed as a potential conflict of interest.

## Publisher's Note

All claims expressed in this article are solely those of the authors and do not necessarily represent those of their affiliated organizations, or those of the publisher, the editors and the reviewers. Any product that may be evaluated in this article, or claim that may be made by its manufacturer, is not guaranteed or endorsed by the publisher.
